# Autonomous countertraction for secure field of view in laparoscopic surgery using deep reinforcement learning

**DOI:** 10.1007/s11548-024-03264-2

**Published:** 2024-09-16

**Authors:** Yuriko Iyama, Yudai Takahashi, Jiahe Chen, Takumi Noda, Kazuaki Hara, Etsuko Kobayashi, Ichiro Sakuma, Naoki Tomii

**Affiliations:** Graduate School of Engineering, The University of Tokyo, Tokyo, Japan

**Keywords:** Reinforcement learning, Countertraction, Autonomous manipulation, Computer-assisted surgery, Minimally invasive surgery

## Abstract

**Purpose:**

Countertraction is a vital technique in laparoscopic surgery, stretching the tissue surface for incision and dissection. Due to the technical challenges and frequency of countertraction, autonomous countertraction has the potential to significantly reduce surgeons’ workload. Despite several methods proposed for automation, achieving optimal tissue visibility and tension for incision remains unrealized. Therefore, we propose a method for autonomous countertraction that enhances tissue surface planarity and visibility.

**Methods:**

We constructed a neural network that integrates a point cloud convolutional neural network (CNN) with a deep reinforcement learning (RL) model. This network continuously controls the forceps position based on the surface shape observed by a camera and the forceps position. RL is conducted in a physical simulation environment, with verification experiments performed in both simulation and phantom environments. The evaluation was performed based on plane error, representing the average distance between the tissue surface and its least-squares plane, and angle error, indicating the angle between the tissue surface vector and the camera’s optical axis vector.

**Results:**

The plane error decreased under all conditions both simulation and phantom environments, with 93.3% of case showing a reduction in angle error. In simulations, the plane error decreased from $3.6 \pm 1.5{\text{ mm}}$ to $1.1 \pm 1.8 {\text{mm}}$, and the angle error from $29 \pm 19 ^\circ$ to $14 \pm 13 ^\circ$. In the phantom environment, the plane error decreased from $0.96 \pm 0.24{\text{ mm}}$ to $0.39 \pm 0.23 {\text{mm}}$, and the angle error from $32 \pm 29 ^\circ$ to $17 \pm 20 ^\circ$.

**Conclusion:**

The proposed neural network was validated in both simulation and phantom experimental settings, confirming that traction control improved tissue planarity and visibility. These results demonstrate the feasibility of automating countertraction using the proposed model.

## Introduction

Laparoscopic surgery is characterized by minimal incisions using forceps along with a laparoscope. It offers several advantages to patients, including smaller surgical wounds, reduced pain, and shorter hospital stays compared to open surgery [1]. However, surgeons experience significant stress when manipulating long forceps within limited movement ranges while viewing the surgical field on monitors [2, 3]. Autonomous surgery has the potential to alleviate the workload and enhance procedural safety and quality [4].

In laparoscopic surgery, applying appropriate tension to tissues and ensuring adequate visibility of the surgical field are crucial for procedures such as incision and dissection [5]. Countertraction, a technique in which an assistant manipulates the forceps to achieve an optimal mechanical stress state of the tissue while maintaining a planar tissue surface face, is indispensable for effective tissue separation. Countertraction accounts for more than 60% of the total surgical time [6]; thus, its automation is expected to significantly reduce the surgeons’ workload.

Autonomous countertraction involves manipulating non-rigid tissues, which have more degrees of freedom than rigid tissues. Previous studies [7–9] have used adaptive linear control based on geometric features to manipulate non-rigid tissues. However, these studies assumed simple linear elastic bodies, raising concerns about their applicability to biological tissues with nonlinear characteristics. Other approaches based on object contours in images [10–12] are unable to perform three-dimensional (3D) shape manipulations.

Recent research has explored the use of reinforcement learning (RL) to address these challenges. RL offers advantages in inferring elastic properties through agent manipulation and automatically optimizing control policies [13, 14]. Thananjeyan et al. [15–17] focused on automating traction to assist pattern cutting using deep RL. Their goal was to maintain the planned incision line during cutting by automatically retracting the tissue with a single pair of forceps, optimizing the grasping position and traction direction to minimize the error between the planned and actual incision lines. However, there are limitations in the formulation of these methods. First, they assumed that the periphery of the rectangular tissue is fixed, maintaining a level of tissue tension that diverges significantly from real surgical conditions. Additionally, the traction direction was restricted to four directions in two dimensions (2D). Therefore, to the best of our knowledge, automation that improves surgical field visibility and applies tension to tissues, as required for countertraction, has not been achieved thus far.

This study aimed to demonstrate the feasibility of using an RL model to control countertraction, enhancing the planarity and visibility of sub-surgical tissues. We proposed a neural network that combines a point cloud convolutional neural network (CNN) with a deep RL model. This network determines the continuous direction and magnitude of forceps traction based on tissue surface point clouds and forceps tip positions. After training the model in a physical simulation environment, we validated its feasibility by applying it to membrane-like tissue phantoms in both simulated and phantom experimental scenarios.

## Methods

In this study, we focused on countertraction during mesenteric dissection in colorectal surgery as a representative scenario. The objective of countertraction during mesenteric dissection was defined as enhancing the planarity and visibility of tissue surfaces to secure the field of view in laparoscopic surgery. Accordingly, the reward functions were designed to reflect this objective. The RL model determines the traction direction and magnitude to maximize the expected rewards based on the observed point cloud of the membrane-like tissue surfaces and the current position of the forceps tip.

### Observation and action space

In this study, we defined the observed state $s_{t}$ from the environment, which serves as the input to the RL model, as follows:

1 $$ \begin{array}{*{20}c} {s_{t} = \left( {\overrightarrow {{p_{1} }} \left( t \right), \ldots , \overrightarrow {{p_{N} }} \left( t \right), \overrightarrow {{g_{1} }} \left( t \right), \overrightarrow {{g_{2} }} \left( t \right)} \right)} \\ \end{array} $$

where $\overrightarrow {{p_{i} }} = \left( {x_{i} , y_{i} , z_{i} } \right), i \in \left[ {1,N} \right]$ represents the coordinate of its point in the point cloud of the tissue surface, and $\overrightarrow {{g_{j} }} = \left( {x_{j} , y_{j} , z_{j} } \right), j \in \left[ {1,2} \right]$ denotes the tip positions of the two forceps, respectively. Assuming that the transformation matrix between the robot arm and camera can be obtained through hand-eye calibration, both $\overrightarrow {{p_{i} }}$ and $\overrightarrow {{g_{j} }}$ are defined as values in the camera coordinate system.

The output from the RL model, denoted as action $a_{t}$, is defined as the displacement of the forceps tips in the camera coordinate system. This action $a_{t}$ is obtained by applying a transformation matrix $T_{r}^{c}$ to convert the camera coordinate system to the robot coordinate system. Here, $\Delta \overrightarrow {{g_{k} }} = \left( {\Delta x_{k} , \Delta y_{k} , \Delta z_{k} } \right), k \in \left[ {1,2} \right]$.

2 $$ \begin{array}{*{20}c} {a_{t} = {\text{T}}_{{\text{r}}}^{{\text{c}}} \left( {\Delta \overrightarrow {{g_{1} }} \left( t \right), \Delta \overrightarrow {{g_{2} }} \left( t \right)} \right)} \\ \end{array} $$

### Reward function

The reward function quantifies the desirability of the performed action, calculating a reward value based on the environment. We quantitatively evaluated the shape and orientation of the tissue surface using a point cloud representation. For the surface shape, closer proximity to a plane indicated better planarity. Consequently, we evaluated the planarity by calculating the distance between the 3D point cloud of the tissue surface and the least-squares plane. Let $\vec{h}$ denote the unit normal of the least-squares plane and $\overrightarrow {{p_{G} }}$ be the centroid of the 3D point cloud $\left( {\overrightarrow {{p_{1} }} , \ldots , \overrightarrow {{p_{N} }} } \right)$. The average distance $\underline {d}$ between the tissue surface and the least-squares plane was determined as follows:

3 $$ \begin{array}{*{20}c} {\overline{d} = \frac{1}{N}\mathop \sum \limits_{i = 1}^{N} \left| {\left( {\overrightarrow {{p_{i} }} - \overrightarrow {{p_{G} }} } \right) \cdot \vec{h}} \right|} \\ \end{array} $$

For surface orientation, we assumed that better visibility was ensured when the tissue surface faces toward the camera’s optical axis direction. We evaluated this by the cosine value of the angle $\theta$ between the normal vector of the least-squares plane and the camera optical axis vector $\vec{c}$. The cosine value was performed as follows:

4 $$ \begin{array}{*{20}c} {\cos \theta = \frac{{\vec{c} \cdot \vec{h}}}{{\left| {\vec{c}} \right|\left| {\vec{h}} \right|}}} \\ \end{array} $$

The reward value for the RL model $R$ was calculated by integrating the assessments of the surface shape and orientation, with regularization parameters $a$ and $b$.

5 $$ \begin{array}{*{20}c} {R = \left( {\frac{2}{{\exp \left( {\overline{d}} \right) + 1}}} \right)^{a} \left( {\cos \theta } \right)^{b} } \\ \end{array} $$

Furthermore, to penalize excessive tissue deformation, negative rewards were assigned based on the strain values between particles. The distance $d_{ij}^{t}$ between points $i$ and $j$ in the 3D point cloud at time $t$, denoted by ( $\overrightarrow {{p_{1}^{t} }} , \ldots , \overrightarrow {{p_{N}^{t} }}$), can be calculated as follows:

6 $$ \begin{array}{*{20}c} {d_{ij}^{t} = \left| {\left| {\overrightarrow {{p_{i}^{t} }} - \overrightarrow {{p_{j}^{t} }} } \right|} \right|} \\ \end{array} $$

At each time $t$, the maximum strain between each pair of points was defined as the maximum interpoint strain $\varepsilon_{max}^{t}$. A reward of -1 was assigned when $\varepsilon_{max}^{t}$ exceeded a certain threshold.

7 $$ \begin{array}{*{20}c} {\varepsilon_{max}^{t} = \mathop {\max }\limits_{{i,{ }j}} \frac{{\left| {d_{ij}^{t} - d_{ij}^{0} } \right|}}{{d_{ij}^{0} }}} \\ \end{array} $$

### Neural network architecture

**Fig. 1:**
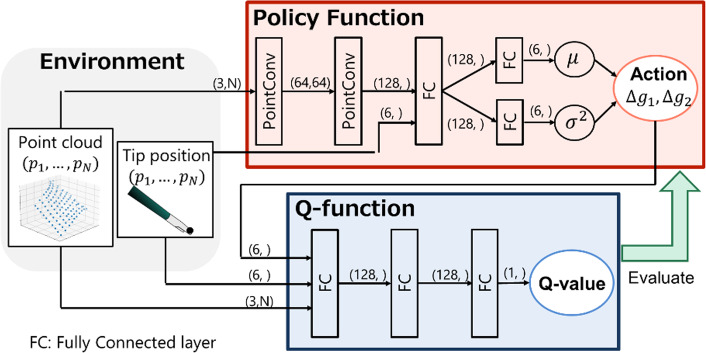
Neural network architecture. The numbers above the arrows represent the dimensions of the inputs. The policy function includes PointConv and fully connected layers, which take the point cloud of the membrane tissue and the end-effector positions (in the camera coordinate system) as inputs and output the displacements of each end-effector position (in the camera coordinate system). The Q-function comprises three fully connected layers

Figure 1 illustrates the neural network structure used in this study. To enable continuous control in any direction, we developed a machine-learning model based on a soft actor-critic [18], which is capable of handling continuous actions. The soft actor-critic (SAC) consists of a policy function that outputs actions from states, a Q-function that outputs Q-values from states, and actions and rewards calculated by the reward function. The policy function determines the traction direction, whereas the Q-function evaluates and updates this direction. We utilized the mean squared error (MSE) as a loss function to update the Q-function and entropy-regularized policy loss to update the policy, same as the SAC model [18]. In our approach, we employed PointConv [19], a point cloud CNN, for the policy function neural network instead of the traditional fully connected layers. PointConv is a neural network that maintains permutation invariance and manages the relationship between neighboring points in point clouds, thus facilitating effective feature extraction from non-rigid tissue shapes.

## Simulation experiments

In deep RL, accumulating a significant amount of data requires trial and error, which complicates real-world learning due to time and safety constraints. Therefore, we conducted learning within a simulated environment and carried out verification experiments.

### Physics simulator

**Fig. 2:**
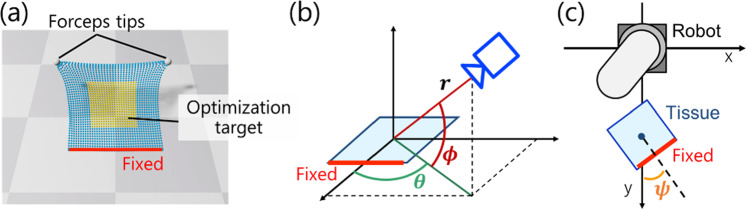
Physical simulation environment and definition of each angle. **a** depicts the physical simulation environment. The yellow area represents the optimization target, the red area indicates the fixed position, the white spheres represent the forceps tips, and the blue part denotes other points on the tissue. **b** defines the orientation of the camera relative to the tissue using the distance $r$, azimuth angle $\theta$, and elevation angle $\phi$. **c** defines the fixed posture angle of the tissue relative to the robot using the rotation angle $\psi$

We established a physical simulation environment using Nvidia Flex [20, 21], which models non-rigid tissues as collections of particles with position constraints based on position-based dynamics [22]. The stiffness coefficients for calculating the elastic potential energy related to stretching, bending, and shearing constraints were set at 0.8, 1.0, and 0.9, respectively. We simulated a 3D non-rigid, surface-shaped tissue, analogous to membranous tissues, such as the mesentery, incorporating forceps tips. The size of the tissue was 0.2 m × 0.2 m, and it was composed of a grid of 40 × 40 points. One edge of the tissue was fixed, and the grasp position was set at the top, targeting optimization at the tissue’s center (Fig. 2a).

### Training condition

We defined the camera position relative to the tissue using the distance $r$, azimuth angle $\theta$, and elevation angle $\phi$, as illustrated in Fig. 2b. Additionally, we defined the tissue’s fixed posture angle relative to the robot using the rotation angle $\psi$ as shown in Fig. 2c. To enable the trained model to dynamically adjust tissue traction based on various camera positions relative to the tissue during surgery, we conducted training across 45 environments by varying $r$ in three ways, $\theta$ in five ways, and $\phi$ in three ways (Table 1).

**Table 1:** The setting of the tissue and camera positions in the simulation environment during training

Distance ($r$ [m])	0.32, 0.40, 0.48
Azimuth angle ($\theta \left[ {deg} \right]$)	− 60, − 30, 0, 30, 60
Elevation angle ($\phi \left[ {deg} \right]$)	30, 45, 60
Rotation angle ($\psi \left[ {deg} \right]$)	0

An episode was defined as comprising 100 actions performed in the environment. After each set of 100 actions, we reset the environment to its initial state and resumed learning. The regularization parameters $a$ and $b$ in Eq. (5) were set to (5, 5), and the threshold of $\varepsilon_{max}^{t}$ in Eq. (7) was set to 5.0. If negative rewards (− 1) were given due to significant tissue deformation, the environment was reset to its initial state at that point. Training was terminated when the total number of episodes reached 9900.

## Evaluation method

Validation experiments were conducted to assess the efficacy of traction in improving the visibility and planarity of the membranous tissue under various camera positions. A total of 300 randomly configured conditions were tested, with distances $r$ ranging from 0.32 to 0.48 m, azimuth angles $\theta$ between − 60 and 60$^\circ$, and elevation angles $\phi$ between 30 and 60$^\circ$. The evaluation was based on comparisons of the surface planarity error ($\underline {d}$.) and angle error ($\theta$) between the initial and final states using Eqs. (3) and (4), respectively.

Furthermore, validation experiments were conducted to assess the feasibility of control under varying tissue-fixed postures relative to the robot. The same 300 conditions were tested, with the rotation angle $\psi$ ranging from 0 to 360$^\circ$ in 30$^\circ$ increments. The evaluation involved comparing the mean and standard deviation of final reward values for each rotation angle $\psi$.

## Phantom experiment

Experiments were conducted using a membrane-like phantom to evaluate the applicability of the model trained in a physics simulation environment to phantom experimental scios. Owing to limitations in the experimental setup, the robotic arm could control only one forceps in the phantom environment. Consequently, the model was retrained, and validation experiments were conducted with other immobilized forceps.

### Experimental setup

**Fig. 3:**
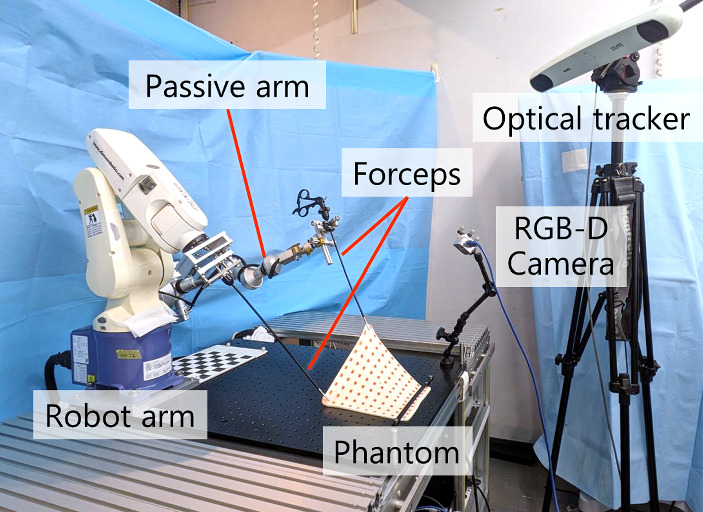
Experimental setup

Figure 3 illustrates the experimental setup. A membrane-like phantom, chosen for its resemblance to human tissue and its ease of shaping, was used (Ekushi-ru Corp., Japan). The phantom measured 0.2 × 0.2 m and 10 × 10 markers was placed on its surface to facilitate its recognition as a point cloud. The phantom was imaged using an RGB-D camera (RealSense D405, Intel Corp., USA) to acquire a point cloud in the camera coordinate system. Simultaneously, the transformation matrix from the robot’s coordinate system to the camera’s coordinate system, measured using an optical tracker (Polaris Spectra Position System, NDI Corp., USA), was used to convert the end-effector position of the forceps, calculated from the robot’s joint angles from the robot coordinate system to the camera coordinate system. Using the obtained 3D point cloud and the end-effector position of the forceps in the camera coordinate system as inputs, the displacement of the forceps end-effector position was determined by the trained model. Based on this displacement, traction control was executed using the robotic arm (VP6242, Denso Corp., Japan). To ensure regular acquisition of the 3D point cloud, markers were applied to the phantom surface.

### Pre-training in simulation

To adapt to the experimental conditions in the phantom environment, the training conditions in the physics simulation were modified, and the model was retrained accordingly. Initially, reflecting the phantom environment where only one forceps was manipulated, the tip of the fixed forceps was positioned at a height of 0.165 m on the Z-axis, while the XY coordinates remained consistent with the original forceps position. Moreover, although the simulation environment facilitated observations with $40 \times 40$ points, it was impractical to observe and process the same number of points in the phantom environment. Therefore, the point cloud observed was reduced to $10 \times 10$ points.

### Evaluation method

Validation experiments were conducted to assess the effectiveness of traction in enhancing the visibility and planarity of membranous tissue under various camera positions. A total of 30 randomly configured conditions were tested, with distances $r$ ranging from 0.32 to 0.48 m, azimuth angles $\theta$ from − 60 to 60$^\circ$, and elevation angles $\phi$ from 30 to 60$^\circ$. The evaluation was based on comparisons of the surface planarity error ($\underline {d}$.) and angle error ($\theta$) between initial and final states using Eqs. (3) and (4), respectively.

Additionally, to evaluate the control’s adaptability when the fixed posture of the organization relative to the robot changed, experiments were conducted with varying rotation angles $\psi$. (0$^\circ$, 90$^\circ$, and 270$^\circ$). Thirty sets of conditions were validated for each rotation angle. The evaluation involved comparing the mean and standard deviation of the final reward values for each rotation angle.

## Results

### Simulation experiment

**Fig. 4:**
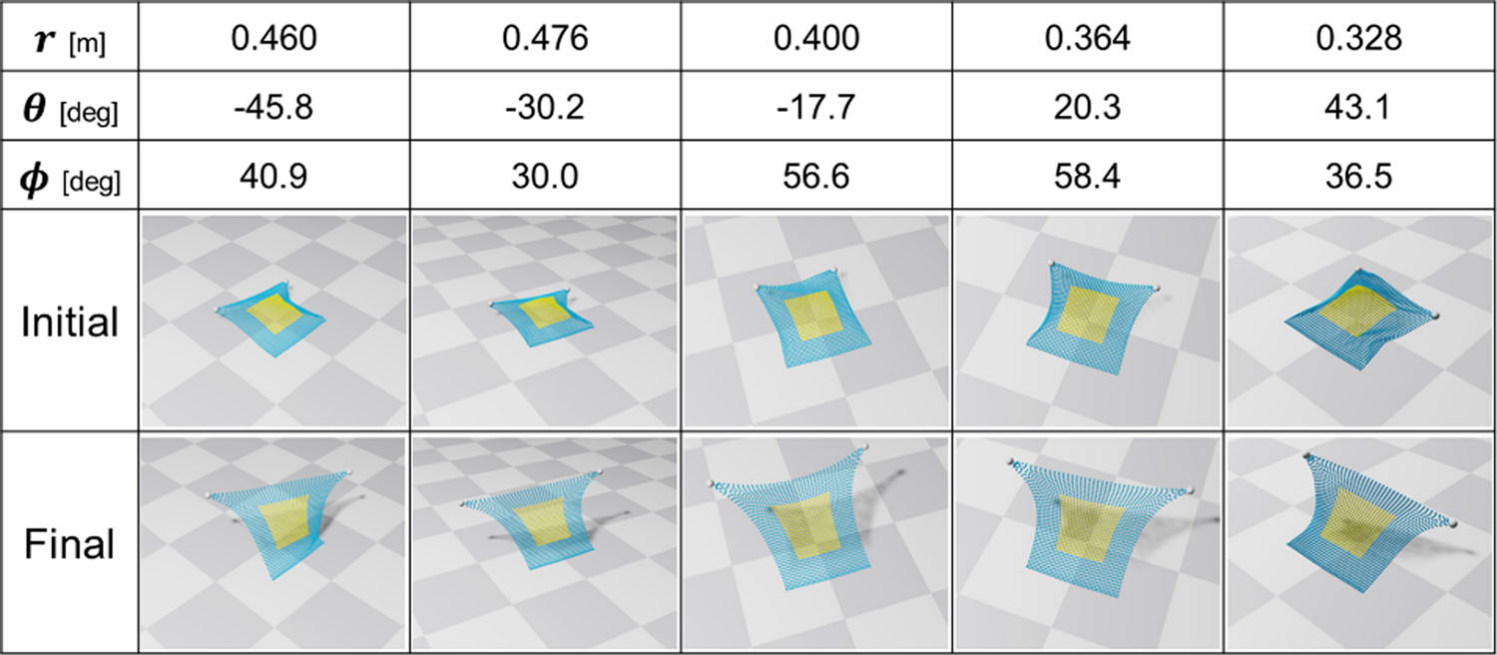
Control results in the simulation environment

**Fig. 5:**
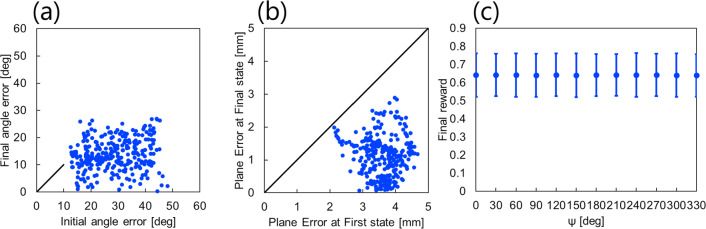
Analysis results in the simulation environment. **a** and **b** depict the angle error and plane for each condition in both the initial and final states, respectively. The horizontal axis represents the values in the initial state, and the vertical axis represents the values in the final state. Points located below the black diagonal line denote a decrease in error, whereas points above it signify an increase. **c** Mean and standard deviation of the final reward values for each rotation angle $\psi$ of the robot

Figure 4 illustrates the control behavior for five selected conditions, each with diverse values for distance $r$, azimuth angle $\theta$, and elevation angle $\phi$ among the 300 conditions used in the validation experiment. Figure 5(a) and (b) displays the plane error ($\underline {d}$ in Eq. (3)) and angle error ($\theta$ in Eq. (4)) for each condition in both the initial and final states. It is evident that the plane error decreased under all conditions, with 93.3% of the 300 conditions experiencing a reduction in angle error. The plane error was initially 3.6 ± 1.5 mm and reduced to 1.1 ± 1.8 mm in the final state. The angle error was initially 29 ± 19$^\circ$ and reduced to 14 ± 13$^\circ$ in the final state.

Figure 5(c) shows the mean and standard deviation of the final reward values for each condition where the fixed posture of the organization relative to the robot changed. As shown, the mean and standard deviation of the final reward values remained consistent across all conditions.

## Phantom experiment

**Fig. 6:**
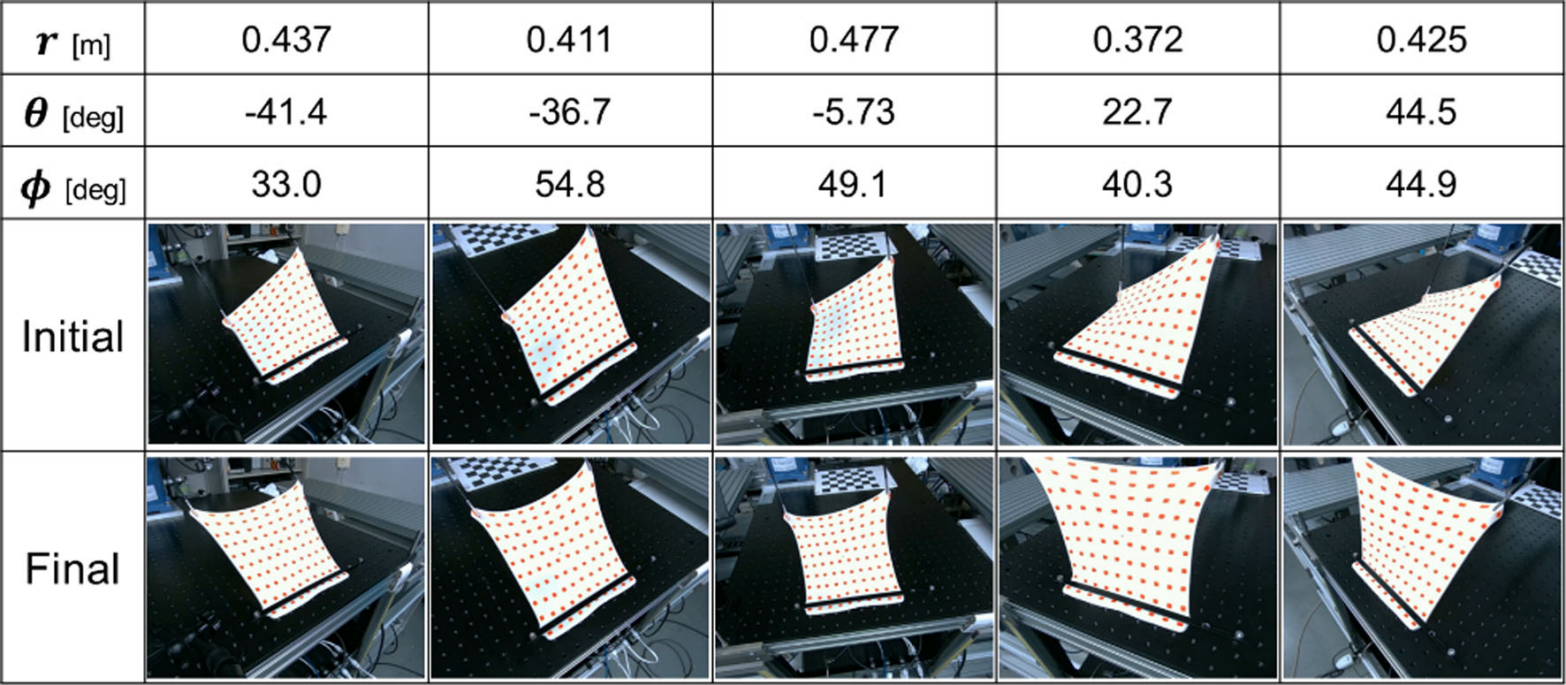
Control result in the phantom experimental environment

**Fig. 7:**
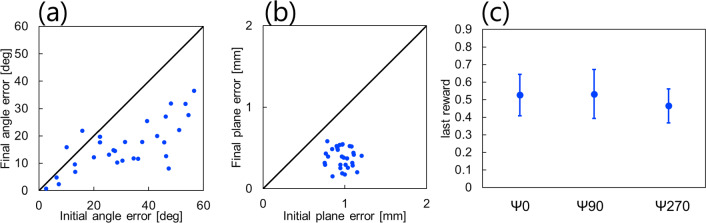
Analysis results in the real environment. **a** and **b** depict the angle error and plane error for each condition in both the initial and final states, respectively. The horizontal axis represents the values in the initial state, while the vertical axis represents the values in the final state. Points located below the black diagonal line denote a decrease in error, whereas points above it signify an increase in error. **c** Mean and standard deviation of the final reward values for each rotation angle $\psi$ of the robot

Figure 6 illustrates the control behavior for five selected conditions, chosen to represent as diverse values as possible for distance $r$, azimuth angle $\theta$, and elevation angle $\phi$ among the 30 conditions used in the validation experiment. In addition, Fig. 7(a) and (b) presents the plane error and angle error for each condition in both the initial and final states, respectively. The plane error decreased in all conditions, with 93.3% of the 30 conditions experiencing a reduction in angle error. Initially, the plane error was 0.96 ± 0.24 mm and reduced to 0.39 ± 0.23 mm in the final state. The angle error was initially 32 ± 29$^\circ$ and reduced to 17 ± 20$^\circ$ in the final state.

Furthermore, Fig. 7(c) displays the mean and standard deviation of the final reward values for each condition where the fixed posture of the organization relative to the robot changed. Across all conditions, the mean and standard deviation of the final reward values were consistent, with a mean final reward value of 0.51 ± 0.043.

## Discussion

This study aimed to demonstrate the feasibility of using an RL model for controlling countertraction to enhance the planarity and visibility of sub-surgical tissues. We proposed a neural network comprising a point cloud CNN and a deep RL model to determine the continuous direction and magnitude of forceps traction based on tissue surface point clouds and forceps tip positions. After training the model in a physical simulation environment, we validated its feasibility by applying it to membrane-like tissue phantoms in both simulated and phantom experimental scenarios. In both environments, the plane error decreased in all conditions, with 93.3% experiencing a reduction in angle error, as shown in Fig. 5a, b and Fig. 7a, b. In the simulation, the plane error decreased from $3.6 \pm 1.5 {\text{mm}}$ to $1.1 \pm 1.8{\text{ mm}}$, and the angle error decreased from $29 \pm 19 ^\circ$ to $14 \pm 13 ^\circ$. In the phantom experimental environment, the plane error decreased from $0.96 \pm 0.24{\text{ mm}}$ to $0.39 \pm 0.23{\text{ mm}}$, and the angle error decreased from $32 \pm 29 ^\circ$ to $17 \pm 20 ^\circ$. Based on these results, we successfully demonstrated the feasibility of using an RL model for controlling countertraction to enhance the planarity and visibility of sub-surgical tissues under the current learning and experimental conditions.

The mean and standard deviation of the final reward values consistently converged to sir values regardless of the fixed angle of the tissue relative to the rob, as illustrated in Figs. 5c and 7c. This suggests that the RL model can effectively learn control strategies for tissue countertraction based solely on visual information.

Although the plane error decreased across all conditions, instances occurred where the angle error did not diminish. An analysis was conducted to clarify the conditions under which the reduction in the angle error was not observed. The simulation environment revealed that instances where the angle error did not decrease were predominantly at elevation angles $\phi$ of 54$^\circ$ or greater, with a concentration in regions where the azimuth angle $\theta$ was 0$^\circ$ or less. Under these conditions, where the angular error did not significantly decrease, the camera position tended to be directly above e tissue, and the initial angle error was smaller compared to other conditions. Since the reward function was designed to reduce both plane and angle errors, it appears that the model prioritized reducing the plane error over the already minimal angle error.

In the phantom experimental environment, where only one grasping point was manipulated, it was revealed that the instances of unimproved angle error were not influenced by the distance $r$ or elevation angle $\phi$ but were concentrated near $\theta = - 60 ^\circ$. This pattern emerged because, in this specific experimental setup with only one fixed pair of forceps, points in proximity to $\theta = - 60 ^\circ$ already exhibited minimal angle errors at the outset. In contrast, evaluation experiments conducted in the simulation environment using two movable forceps did not show a significant increase in angle error around θ =  − 60°. It is postulated that the combined movement of two grasping points allows for more flexible changes in the reward function. Therefore, this phenomenon is likely avoidable not due to ineffective learning but rather through the implementation of dual forceps.

We used an RGB-D camera to extract feature points in the phantom experiment. The number and order of detected points may vary between frames. The integration of PointConv in the feature extraction unit, replacing fully connected layers in the soft actor-critic model, was instrumental in managing these changes.

This study focused on countertraction for mesenteric dissection in colorectal surgery and developed a corresponding simulation environment. Given the critical nature of maintaining planarity through three-dimensional traction for effective countertraction [23], we devised a reward function emphasizing planarity and surface directionality, with a focus on visibility from the camera. This design approach allowed the reinforcement learning model to learn effective control mechanisms tailored to the specific requirements of the procedure. However, it should be noted that the reward function optimized for this scenario may not be universally applicable. For instance, in colorectal surgery, a reward function that prioritizes planarity may become less relevant following the dissection of the membrane. In such cases, automation could be facilitated by adapting the reward function to suit the new context. Additionally, when dissecting the membrane, it is crucial to determine the ideal orientation of the tissue surface by taking into account the position and direction of the dissection device.

In this study, we developed a simulation environment based on the assumption that the target tissue for traction was located near a fixed area, which facilitated the deformation of the target tissue relatively easy. However, in actual surgeries, it is often necessary to manipulate tissues that are farther away from the fixed areas. Countertraction of tissues in such situations is more challenging and it can be considered a current limitation of our model. Therefore, future improvements should focus on enabling the system to handle tissues that are a certain distance away from the fixed areas.

To mitigate the risk of damaging biological tissues, we have introduced a penalty in the model when the separation between points on the tissue surface surpasses a predefined threshold, indicating a manipulation failure under the learning conditions. However, this threshold may not be suitable for all tissue types, and thus cannot fully ensure the avoidance of tissue damage. This limitation presents a significant challenge for clinical applications. Further research into the tolerance levels of real biological tissues under strain is essential. Such studies will guide the development of more refined control strategies that prevent tissue damage.

In this study, the RL model accepted the 3D point cloud of tissues as input, assuming accurate acquisition of tissue surface shapes. Although markers have been used in phantom experiments, their use in the actual surgery of biological tissues is challenging. Hence, exploring markerless methods for obtaining 3D point clouds of biological tissues remains essential for applying the proposed method in surgical settings. Given that various studies have explored markerless methods for this purpose [24, 25], integration with such technologies will be essential in the future.

## Conclusions

This study proposed a neural network that integrates a point cloud CNN with a deep RL model, aimed at automating countertraction to reduce the workload on surgeons in laparoscopic procedures, offering an alternative to manual assistance. The model was designed to generate continuous displacements for two forceps based on the 3D point cloud data of tissue surfaces and the positional data of the forceps tips. A specifically designed reward function targeted the enhancement of tissue surface planarity and visibility by taking into account the shape and orientation of the surface. The proposed neural network was validated in both a PBD-based simulation environment and a phantom experimental setting, confirming that traction control significantly improved the planarity and visibility of the membranous tissue in both scenarios. These results demonstrated the feasibility of automating countertraction using the proposed model.
